# AMP-regulated protein kinase activity in the hearts of mice treated with low- or high-fat diet measured using novel LC–MS method

**DOI:** 10.1007/s11010-015-2360-z

**Published:** 2015-02-25

**Authors:** I. M. Rybakowska, E. M. Slominska, P. Romaszko, M. Olkowicz, K. Kaletha, R. T. Smolenski

**Affiliations:** 1 Department of Biochemistry and Clinical Physiology, Medical University of Gdansk, Debinki 1, 80-211 Gdańsk, Poland; 2 Department of Biochemistry, Medical University of Gdansk, Gdańsk, Poland; 3 Department of Biotechnology and Food Microbiology, Poznan University of Life Sciences, Poznan, Poland

**Keywords:** AMP-regulated protein kinase, AMP-deaminase, Adenosine deaminase, High-fat diet

## Abstract

AMP-regulated protein kinase (AMPK) is involved in regulation of energy-generating pathways in response to the metabolic needs in different organs including the heart. The activity of AMPK is mainly controlled by AMP concentration that in turn could be affected by nucleotide metabolic pathways. This study aimed to develop a procedure for measurement of AMPK activity together with nucleotide metabolic enzymes and its application for studies of mice treated with high-fat diet. The method developed was based on analysis of conversion of AMARA peptide to pAMARA by partially purified heart homogenate by liquid chromatography/mass spectrometry (LC/MS). Activities of the enzymes of nucleotide metabolism were evaluated by analysis of conversion of substrates into products by HPLC. The method was applied for analysis of hearts of mice fed 12 weeks with low- (LFD) or high-fat diet (HFD). The optimized method for AMPK activity analysis (measured in presence of AMP) revealed change of activity from 0.089 ± 0.035 pmol/min/mg protein in LFD to 0.024 ± 0.002 in HFD. This coincided with increase of adenosine deaminase (ADA) activity from 0.11 ± 0.02 to 0.19 ± 0.06 nmol/mg tissue/min and decrease of AMP-deaminase (AMPD) activity from 1.26 ± 0.35 to 0.56 ± 0.15 nmol/mg tissue/min for LFD and HFD, respectively. We have proven quality of our LC/MS method for analysis of AMPK activity. We observed decrease in AMPK activity in the heart of mice treated with high-fat diet. However, physiological consequences of this change could be modulated by decrease in AMPD activity.

## Introduction

AMP-regulated protein kinase (AMPK) is involved in the regulation of energy consumption and fuel generation paths in response to the need of organs such as the liver, central nervous system, adipose tissue, skeletal muscle, and cardiac muscle [1]. AMPK could be activated by AMP by several mechanisms. One of them is allosteric activation of the phosphorylated enzyme; the second is promotion of phosphorylation of Thr-172 by the upstream kinase and the third is the inhibition of dephosphorylation of Thr-172 by protein phosphatases [2]. AMPK acts multidirectionally and some reports indicate a novel biological actions of AMPK which is closely associated with cardiovascular disease. The enzyme is involved in the processes that contribute to morbidity and mortality associated with myocardial ischemia [3]. Pharmacological activation of AMPK in the hypothalamus increases food intake and so AMPK is identified as a target for anti-obesity and anti-diabetic drugs [4, 5]. Some commonly used drugs such as metformin activate AMPK in cardiomyocytes [6]. Another activator of AMPK is a precursor of nucleotide synthesis AICAR (5-aminoimidazole-4-carboxyamide riboside). Indirect activation of AMPK could be achieved by inhibition of AMP degradation e.g., inhibition of AMP-deaminase (AMPD) [7].

**Fig. 1:**
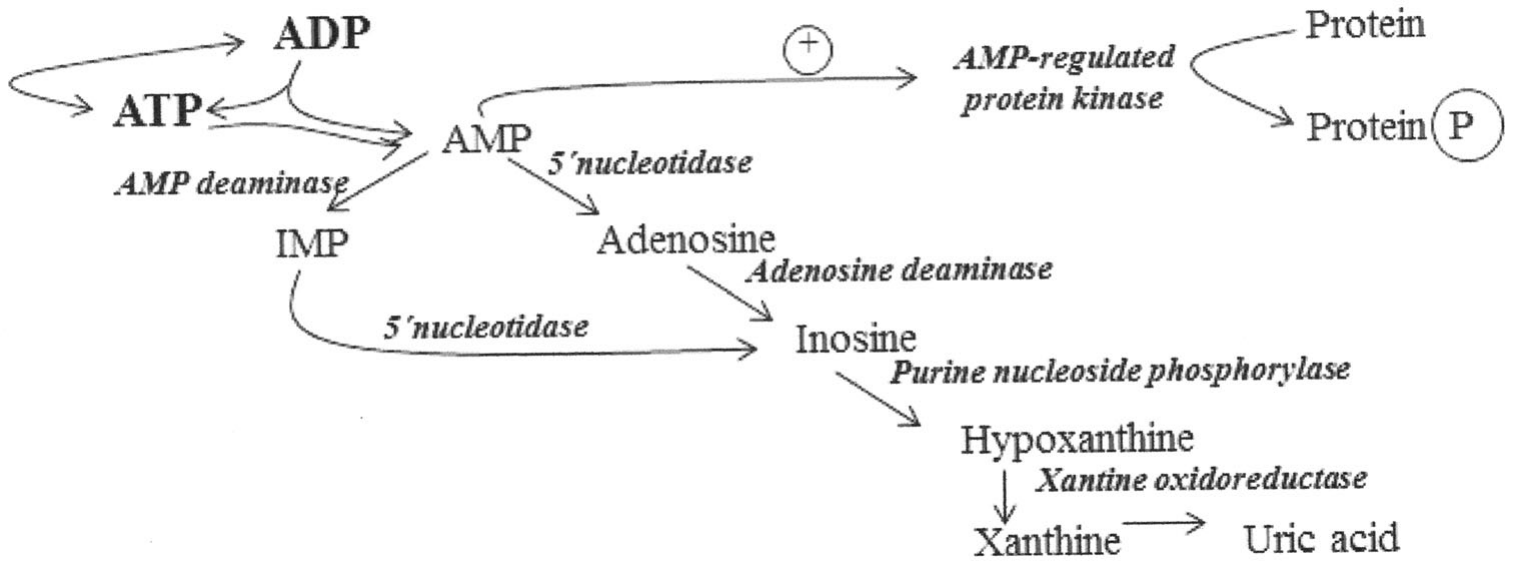
Relation between AMP-regulated protein kinase and adenine nucleotides degradation in heart (modified from M. Żydowo [8])

In cardiac muscle, the physiological role of the reaction catalyzed by AMP-deaminase and also by adenosine deaminase (ADA) is the regulation of adenine and guanine nucleotides pools in the cell [8]. Degradation of AMP by AMP-deaminase changes balance of the reaction catalyzed by myokinase toward production of ATP from ADP nucleotides which helps restore energy equilibrium (Fig. 1). While the reaction catalyzed by AMP-deaminase in human cardiomyocytes during ischemia is less intense than AMP conversion to adenosine, the contribution of AMP-deaminase remains significant [9].

## Materials and methods

### Animals

The study was approved by Medical University of Gdansk Ethics Committee for the Animal Experiments––(29/2012). Three-month-old female mice C57BL6 were fed low-fat diet (DIO Series Diet- D12450B; 20 kcal % protein, 70 kcal % carbohydrate, 10 kcal % fat) or high-fat diet (DIO Series Diet- D12492; 20 kcal % protein, 20 kcal % carbohydrate, 60 kcal % fat) for 12 weeks. The aim of this diet was create model of mice obesity and hyperglycemia. During experiment, mice were weighed and at the end blood glucose was measured. Animals were anesthetized with a mixture of 2.5 µg xylazine/43 µg ketamine per g weight of mice, intubated, and ventilated with a rodent respirator. After opening the chest, the hearts of the mice were frozen with aluminum blocks, precooled in liquid nitrogen, and stored at −80 °C.

### Enrichment of heart homogenate for AMP-regulated protein kinase

Homogenization buffer (HB) consisted of 250 mM mannitol, 50 mM sodium fluoride, 5 mM sodium pyrophosphate, 1 mM benzamidine, 1 mM dithiothreitol, 1 mM EGTA, 1 mM EDTA, 1 mM phenylmethylsulfonyl fluoride, 4 µg/ml soybean trypsin inhibitor, and 50 mM Tris–HCl, pH 7.5. Resuspension buffer (RB) consisted of 50 mM sodium fluoride, 5 mM sodium pyrophosphate, 1 mM benzamidine, 1 mM dithiothreitol, 1 mM EGTA, 1 mM EDTA 1 mM phenylmethylsulfonyl fluoride, 4 µg/ml soybean trypsin inhibitor, 0.02 % sodium azide, 10 % glycerol, and 100 mM Tris–HCl, pH 7.5.

About 20 mg frozen heart was used for AMPK extraction. The tissues were suspended in 5 volume of HB and homogenized at 4 °C. Partial purification of AMPK was carried out according to the modified method of Baron [10] shortly described by our team [11]. After centrifugation of homogenate at 14,000 rpm for 20 min (4 °C), the supernatant was mixed with polyethylene glycol 8000 (PEG) to 2.5 % PEG solution. The solution was mixed for 10 min (4 °C), and after centrifugation at 10,000 rpm for 10 min the resultant supernatant was made up to 6 % PEG. Samples were again mixed for 10 min and centrifuged at 10,000 rpm for 10 min. The 6 % precipitate was collected by centrifugation at 10,000 rpm for 10 min and resuspended in RB, in the same volume as HB.

### Measurement of AMPK activity

Incubation buffer consisted of HEPES mixture pH 7.0 (160 mM NaCl, 80 mM HEPES–NaOH, 1.6 mM EDTA, 16 % glycerol), 2.5 mM AMARA peptide, 100 mM DTT, and 2.5 mM AMP. Buffers without AMP and without substrate were used as controls. The substrate for this reaction was AMARA peptide (AMARAASAAALARRR). Heart AMPK activity was assessed by measuring the resulting phosphorylated form of AMARA. The final substrate concentration for AMPK was 200 µM AMARA; and for other components, the final substrate concentrations were as follows: 40 mM HEPES–NaOH, 80 mM NaCl, 8 % glycerol, 0.8 mM EDTA, 0.8 mM DTT, and 200 µM AMP in tube. Each sample contained ATP mixture (5 mM ATP, 100 mM MgCl_2_, H_2_O). The reaction was initiated by addition of 2 µl of enzyme. The total volume of reaction mixture was 25 µl of which 2.5 µl was ATP mixture with a final concentration 200 µM ATP, 5 mM MgCl_2_. The reaction was carried out for 5 min at 30 °C and was stopped by adding 0.6 % trifluoroacetic acid (TFA) at a final concentration of 0.1 %. Analysis was performed with Surveyor liquid chromatograph connected to the TSQ Vantage triple quadrupole (Thermo Finnigan) equipped with heated electrospray ionization (HESI). The chromatographic separation was carried out on a Phenomenex 3 µm C18 (50 × 0.3 mm) column, maintained at 35 °C with a mobile phase consisting of 0.1 % formic acid (A) and 0.1 % formic acid in acetonitrile (B) at a flow rate 20 µl/min obtained by a flow splitter. A gradient elution program was conducted for chromatographic separation with mobile phase A and B as follows: 0–1.1 min (20–90 % B), 1.1–1.2 min (90–20 % B), 1.2–4.5 min (20–20 % B).

Protein was measured by direct detect IR spectrometer method (Merck-Millipore)

### Analysis of nucleotide enzyme activities

The activities of adenosine deaminase (ADA), AMP-deaminase (AMPD), purine nucleoside phosphorylase (PNP), and ecto-5*′*-nucleotidase (e5NT) were measured by monitoring the formation of the reaction products with HPLC as described before [12]. Hearts were homogenized at 4 °C in 9 volume of buffer (150 mM KCl, 20 mM TRIS, 1 mM EDTA, 1 mM dithiothreitol, pH 7.0). A portion of crude homogenate was taken for determination of e5NT. Remaining homogenate was centrifuged at 3400 rpm at 4 °C for 20 min which was then added in volume 20 µl to the appropriate buffer and was incubated with 20 µl of substrate at the 37 °C. The substrate concentrations were 25 mM AMP for AMPD, 1 mM adenosine for ADA, 1 mM inosine for PNP, and 0.2 mM AMP for e5NT. The reaction was terminated by adding 1.3 M HClO_4_ and subsequently neutralized with 3 M K_3_PO_4_. Centrifuged perchloric acid extracts were analyzed by HPLC [13].

### Statistical analysis

Data presented are expressed as mean ± standard deviation (SD). Enzyme activities were compared by Student’s *t* test. *p* < 0.05 was considered as a significant difference.

## Results

### Animals

**Fig. 2:**
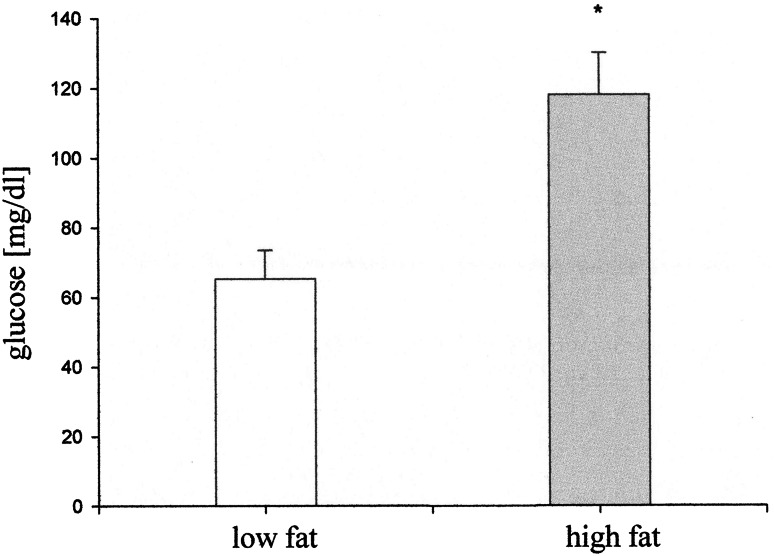
Blood glucose concentration after 12 weeks of feeding mice with low- or high-fat diet. Values are mean ± SD, *n* = 5, *p < 0.05

**Fig. 3:**
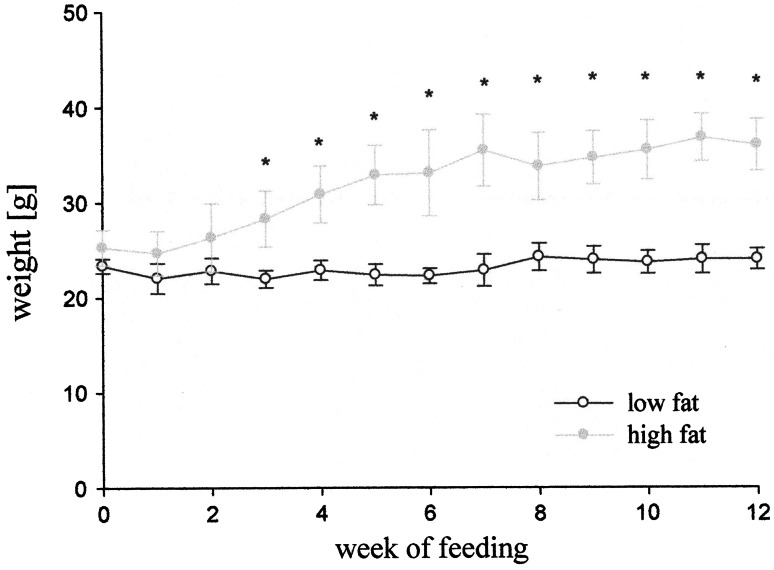
Body weight during experiment in mice fed with high- or low-fat diet. Values are mean ± SD, *n* = 5. *p < 0.05, statistical significance of differences compared to mice fed with low fat at the same time of experiment

Blood glucose in mice fed with high-fat diet was significantly higher than in mice fed with low-fat diet in the twelfth week of feeding (Fig. 2). Statistically significant differences in the weight of animals appeared in the fourth week of diet treatment. Mice fed with high-fat diet increased weight by about 50 % as compared to the initial weight of the mice, whereas the weight of mice fed with low-fat diet remained unchanged (Fig. 3).

### Analysis of AMPK activity

**Fig. 4:**
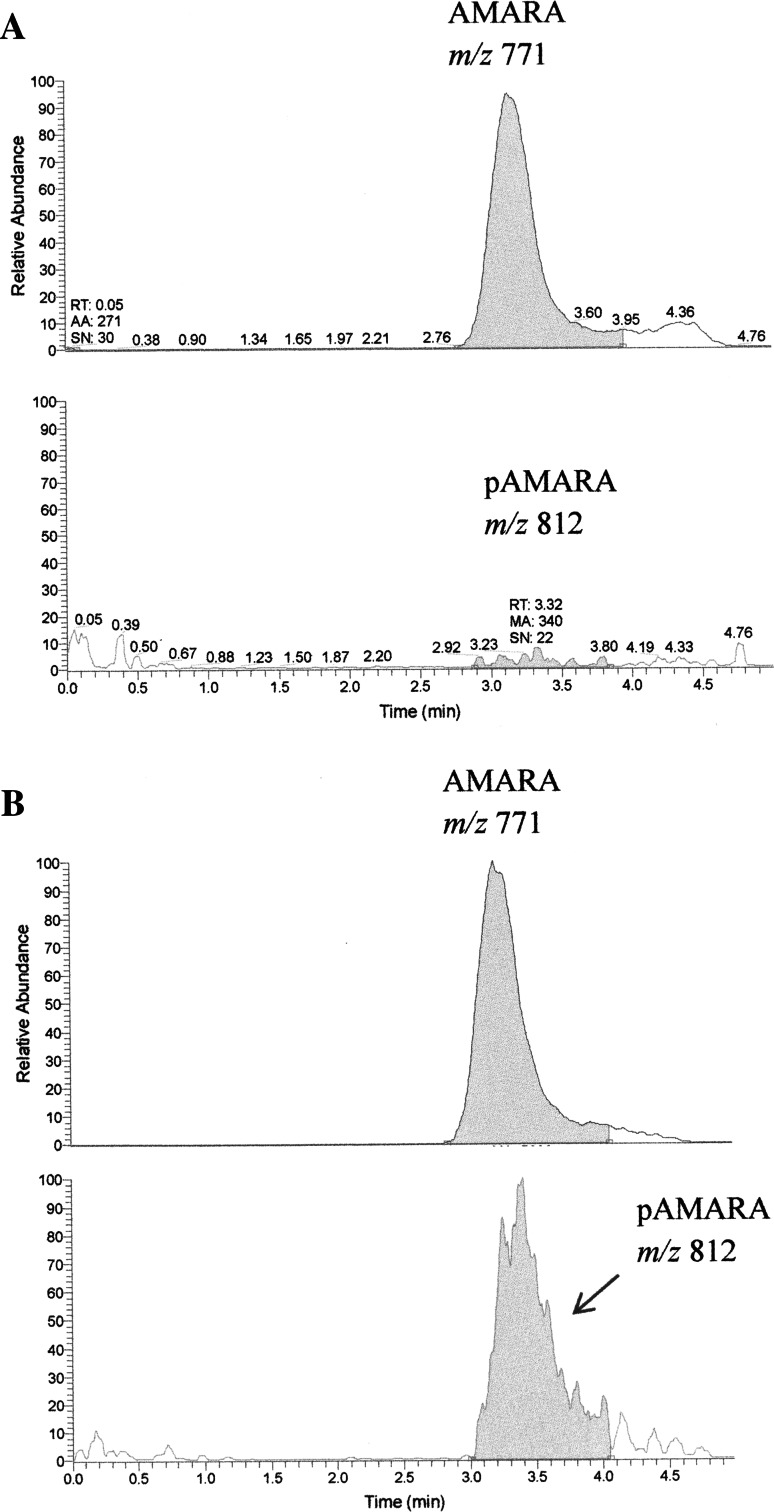
Representation of the extracted ion chromatograms of AMARA and phosphorylated AMARA both as double charged ions; **a** at the beginning of the reaction and **b** at the fifth minute of the reaction

**Fig. 5:**
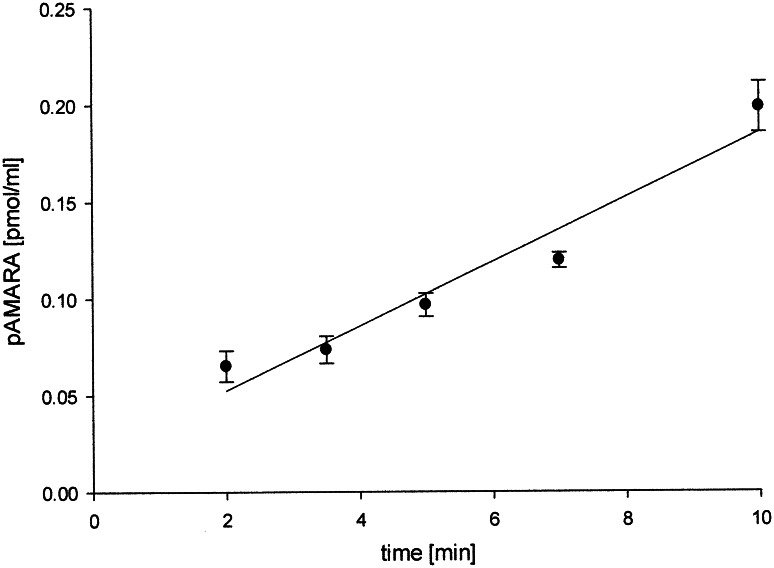
Amount of pAMARA versus time during incubation of fraction mouse heart AMPK. Values are mean ± SD, *n* = 3. Regression for concentrations of pAMARA is *y* = 0.01665*x* + 0.01913

**Fig. 6:**
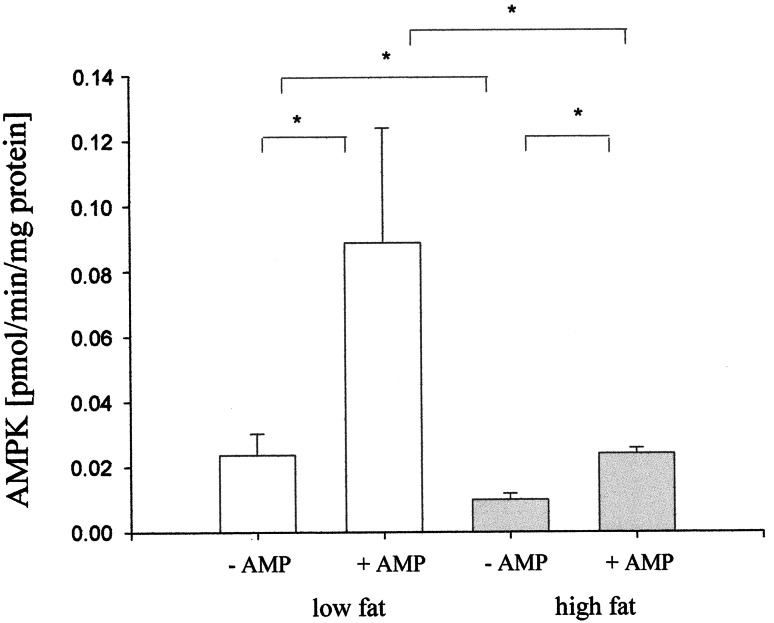
AMPK activity of the mouse heart in the presence or absence of AMP after 12 weeks of feeding with low- or high-fat diet. Reaction was carried out for 5 min with 0.2 mM substrate (AMARA). AMP was present at 0.2 mM concentration. Values are mean ± SD, *n* = 3, *p < 0.05

At the zero time, we did not observe any product of reaction in the extracted ion chromatograms (Fig. 4a), while clear peak of product was observed after 5 min of the reaction (Fig. 4b). Formation of phosphorylated AMARA (pAMARA) was linear with regard to time up to 10 min (Fig. 5). Therefore, optimum 5-min duration was selected for further study. AMPK activity analyzed three times in the same heart revealed high reproducibility of the assay with coefficient of variation equivalent to 6.3 %. In the presence of AMP, activity of AMPK increased about fourfold as compared to absence of AMP in mice on low-fat diet. In the absence of AMP, activity of AMPK in mice fed with low-fat diet increased about 50 % as compared to mice fed with high-fat diet (Fig. 6).

### Enzymes involved in the metabolism of adenine nucleotides

**Fig. 7:**
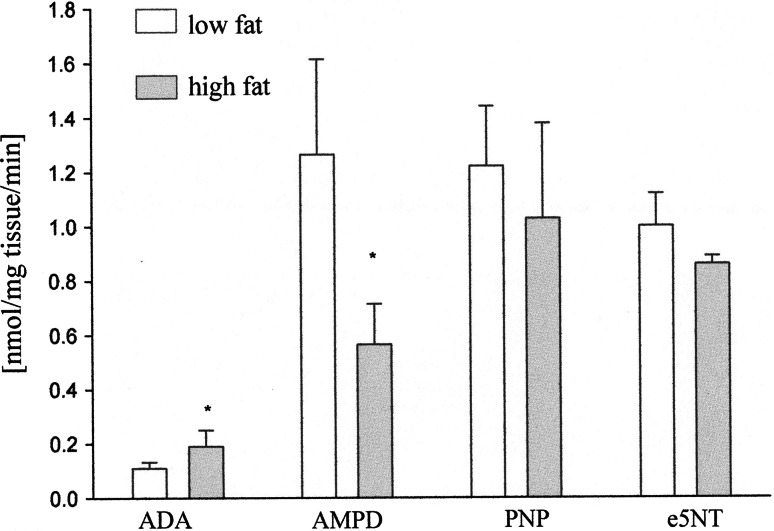
Effect of low- and high-fat diet on the enzymes activities involved in adenine nucleotide metabolism in the mice hearts; *ADA* adenosine deaminase, *AMPD* AMP-deaminase, *PNP* purine nucleoside phosphorylase, *e5NT* ecto-5′-nucleotidase. Values are mean ± SD, *n* = 5, *p < 0.05

AMPD activity was 50 % lower in the hearts of mice fed with high-fat diet. Activity of ADA was two times higher in high-fat diet group. Activities of PNP and e5NT remained unchanged in the hearts of mice in both groups (Fig. 7).

## Discussion

This work demonstrated that LC/MS method which we established could be effectively used for the determination of AMP-regulated kinase (AMPK) in small specimens such as mouse heart and is an effective alternative for radioactive isotopes or antibodies-based procedures. Our studies demonstrated that the activity of AMPK in cardiac muscle could be several times higher in mice fed with low-fat diet compared to high-fat diet. This is consistent with the trend demonstrated in male mice at 6 week of high-fat diet treatment [11]. The changes in AMPK activity observed at 12 week of mice fed with high-fat diet correlates with elevation of blood glucose level. Increase in body weight started from the fourth week of the experiment to 12 week of experiment.

Current methods for analysis of AMPK are burdened by disadvantages such as need of isotopes or antibodies in Western blot and Elisa method. This includes need for special precautions and inconvenience with use of radioactive substances. Use of antibodies introduces the risk of cross reactivity that may alter specificity of the assay. Considering these limitations, we aimed to developed a procedure that will directly follow progress of the reaction. This is possible with use of liquid chromatography linked to mass spectrometry that allows to specifically trace formation of the product of peptide phosphorylation reaction. Besides, high sensitivity of LC/MS may provide additional advantage of reduced need for sample material that is sometimes critical when working with small animals such as mouse.

Maintaining adequate ATP/ADP ratio is essential for cell survival. Therefore, processes that help to maintain energy balance such as AMPK cascade are of prime importance. However, the enzymes involved in the metabolism of nucleotides could modulate function of AMPK. One example is degradation of AMP by AMPD. We investigated, therefore, the activities of the other enzymes involved in the metabolism of adenine nucleotides such as AMPD, ADA, e5NT, and PNP. There were no changes in the PNP and e5NT activities in different diet groups, whereas we observed lower activity of ADA and higher of AMPD activity in mice on low-fat diet compared to high-fat diet. This indicate that despite of low pmoles cardiac activity of AMPK its function may not be so important maybe concurrent decrease of the activity of cardiac AMPD is more significant for mice on high-fat diet. Moreover, our results demonstrated higher activity of ADA in the heart of mice on low-fat diet that may lead to lower concentration of adenosine in hearts of these mice.

In this study, in addition to the new method for determination of AMPK, which may be a good basis for the development of other protein kinase, we provided information on how high-fat diet leading to high glucose concentration and increased body weight affects activity of AMPK and enzymes of nucleotide metabolism. Besides its global effects, we demonstrated that high-fat diet decreases the activity of AMPK in the heart of mice. This could adversely affect cardiac metabolism and in particular its resistance to ischemia. However, lower activity of AMPK could be compensated by concurrent decrease in AMPD activity.
